# Investigating diet and physical activity in Malaysia: education and family history of diabetes relate to lower levels of physical activity

**DOI:** 10.3389/fpsyg.2014.01328

**Published:** 2014-12-03

**Authors:** Cai Lian Tam, Gregory Bonn, Si Han Yeoh, Chee Piau Wong

**Affiliations:** ^1^Jeffrey Cheah School of Medicine and Health Sciences, Monash University MalaysiaBandar Sunway, Malaysia; ^2^Takai Lab, Graduate School of Education and Human Development, Nagoya UniversityNagoya, Japan; ^3^Foreign Research Fellow, Japan Society for the Promotion of ScienceTokyo, Japan

**Keywords:** diabetes, health knowledge, lifestyle, exercise, diet, Malaysia

## Abstract

The National Health and Morbidity Survey (NHMS, 2011), estimates that the number of Malaysian adults suffering from type 2 diabetes has increased from 8.3 to 31.2% since 1996. This study is a preliminary investigation of possible factors contributing to this epidemic. Knowledge of diabetes, health locus of control, diet and exercise habits, as well as family history, education level and other demographic factors to better understand the correlates of risky and healthy behaviors. This was done as part of a larger initiative to improve prevention efforts. Questionnaires were completed by 770 individuals from three Malaysian states: Selangor, Penang, and Terengganu. Findings showed that people with better health knowledge and those who have a family history of type 2 diabetes were more likely to have healthy diets. Also, health knowledge related to lower alcohol consumption. Participants with diabetic family members, however, also reported higher levels of stress. Counterintuitively, higher educational levels, higher internal locus of control, better health knowledge, as well as a family history of diabetes all correlated with lower levels of physical activity. Thus, it is suggested that, while increasing health knowledge will be important in addressing the type 2 diabetes epidemic in Malaysia, especially in relation to diet, other cultural factors, specifically norms related to exercise and physical activity, also need to be addressed if the spread of type 2 diabetes is to be addressed over the long term.

## INTRODUCTION

Type 2 diabetes mellitus, a metabolic disorder, is spreading at epidemic proportions in Malaysia. Generally increases in type 2 diabetes rates are attributed to sedentary lifestyles, unhealthy eating habits (e.g., diets heavy in sugar, oils, and salt), and smoking (Wild et al., 2004). Diabetes (hereafter, the term “diabetes” refers specifically to type 2 diabetes unless otherwise indicated), as a disease, not only effects those suffering from it, but it creates enormous costs to society: it is estimated that direct medical expenses incurred by diabetics are five times higher than those by non-diabetics (Baldacci et al., 2004). The Ministry of Health estimates that, in 2011 alone, Malaysia spent RM18,000,000,000 (about $5.4 billion USD) treating non-communicable diseases (of which diabetes is the most prevalent). Thus, diabetes is set to create a massive burden on public healthcare services if its spread is allowed to continue unchecked. Fortunately, type 2 diabetes is largely preventable and manageable. If healthier lifestyles can be increased among the general population as well as among those with family histories of diabetes it will slow the spread of this preventable disease and greatly reduce its burden on society (NSPNCD, Omar, 2011). Research has shown that risk factors for type 2 diabetes vary in prevalence within Malaysia by geographic area (Nuur Amalina et al., 2012), and ethnicity. Malaysians of Indian ethnicity, for example, have been found to have a greater tendency toward metabolic syndrome and obesity than either Malays or Chinese, while Malays are more likely to suffer from hypertension (Tan et al., 2011; Wee et al., 2011; Rampal et al., 2012). It is not known, however, why these differences exist; or, to what degree knowledge, attitudes, and behaviors vary from group to group. Because groups within Malaysia likely vary in perceived control over their health (i.e., locus of control) and lifestyle, as well as in health knowledge and other beliefs (e.g., Bonn and Tafarodi, 2013), this study explores how these factors interrelate within Malaysian society and its’ component ethnic groups.

### BACKGROUND

Of particular concern is in Malaysia is type 2 diabetes’ increasing prevalence among younger age cohorts: the Ministry of Health estimates that, at current rates, by 2020, 22% of all Malaysians over 18 will suffer from diabetes. Type 2 diabetes being a leading cause of acquired blindness, cardiovascular disease, and kidney failure, as well as a risk factor for cancer, chronic respiratory diseases, and stroke (NSPNCD, Omar, 2011), over time, this will place enormous pressure on the public health care system (see Baptiste-Roberts et al., 2007), possibly amounting to over 25% of the annual health care budget (e.g., Rahman, 2012). Identifying the roots of this disease’s rapid spread, thus, is a high priority from both public health and economic perspectives.

Although the onset of type 2 diabetes can be delayed and even prevented through the adoption of relatively simple lifestyle changes such as increasing physical activity, reducing intake of fats and refined sugars, and moderating use of tobacco and alcohol (Geiss et al., 2010; Chang et al., 2011), until now we haven’t identified exactly what the most salient risks are for various groups within Malaysia and thus how to best focus our prevention efforts. Are people not aware of the factors that contribute to type 2 diabetes and other non-communicable diseases? Are they aware of their unhealthy behaviors, but somehow feel helpless to change? Are there certain resources or knowledge that various groups are lacking which would enable them to live healthier lifestyles? Also, given that Malaysia is a multicultural society comprised of Malays, Chinese, Indians as well as numerous indigenous tribes, each with its own language and distinctive customs, it is important to get some idea of whether the answers to these questions vary by ethnicity (Tan et al., 2011; Wee et al., 2011; Rampal et al., 2012).

#### Health locus of control

Health locus of control describes a person’s characteristic attribution of health outcomes to internal or external causes. According to Rotter (1989), internal versus external locus of control is a personality trait that refers to “the degree to which persons expect that the reinforcement or an outcome of their behavior is contingent on their own behavior or their personal characteristics versus the degree to which persons expect that the reinforcement or outcome is the function of chance, luck, or fate, is under the control of powerful others, or is simply unpredictable.” Lacking of perceived health control permits the growth of fatalistic attributions toward coping with chronic diseases and developing healthy habits (Bailis et al., 2010). A study in China, for example, found that only 26.5% of smokers thought that they could stop smoking (Jiang et al., 2010). Recent research has also linked internal locus of control to healthy eating and exercise habits (Schurer et al., 2012) both of which are key components in the prevention of type 2 diabetes.

#### Health knowledge and healthy behaviors

Although health knowledge does not always predict healthy behaviors (Norris et al., 2001) evidence suggests that knowledge of risks is related to healthy behavioral intentions (Yang et al., 2010). Recent interventions aimed at increasing health knowledge have had some positive results. For example, Fitzpatrick (2011) found that educational programs aimed at adolescents with high blood pressure could be successful at increasing health knowledge, self-efficacy, and readiness for change. Also, Stark et al. (2012) were able to increase healthy behaviors such as exercise and proper nutrition by including a health promotion intervention in an undergraduate course.

#### Family history of diabetes vs. risk factors

Past research focusing on individuals with a family history of type 2 diabetes has provided mixed results. Baptiste-Roberts et al. (2007) found that among African Americans those with a family history of diabetes were more aware of the risk factors, were also more likely to eat fruits and vegetables, made more attempts to lose weight, engaged in more physical exercise, and were more likely to be screened for diabetes compared to others. Similarly, Sultana et al. (2011) found that Pakistanis with a family history of diabetes paid more attention to weight control, amount of physical exercise, and diet as well as kept in more regular contact with their healthcare providers. On the other hand, Rosal et al. (2011) found that low-income, Spanish-speaking participants with a family history of diabetes lacked awareness of risk factors and how to reduce risk in their own lives. Thus, having a family history of diabetes in some cases relates to better health practices, possibly through increased knowledge. Such benefits are not, however, guaranteed. Other factors, such as knowledge and feelings of control may be important mediators in this relationship.

#### Lifestyle and diabetes

Sedentary lifestyle and poor diet are clearly key risk factors for type 2 diabetes (Moore et al., 2011). A study of 113,808 Americans by Bhupathiraju et al. (2013) showed that consumption of sugar-sweetened beverages significantly increased diabetes risk. Janssen (2012) found the greatest risk factor for both men and women in Canada was physical inactivity. Also, regardless of overweight or obesity status, higher intake of fried foods, snacks, and soft drinks, as well as sedentary lifestyle increased the risk of type 2 diabetes while consumption of fruit and vegetables was protective (Bauer et al., 2013). Consuming higher volumes of alcohol per sitting increased risk for both men and women (Crandall et al., 2009; Boggs et al., 2010; Mekary et al., 2011; Heianza et al., 2013). Smoking is also a major risk factor as long-term smoking relates to insulin resistance (Facchini et al., 1992; Targher et al., 1997), possibly even more so among women (Rimm et al., 1993).

#### Knowledge gap

Given the epidemic increase in rates of type 2 diabetes in Malaysia over recent decades, it is crucial to obtain a clearer portrait of exactly what is contributing to this rise, and to use this information to fashion more effective interventions. Most current intervention programs are focused on education, which may be important: Geiss et al. (2010), for example, found that only 7.3% of those sampled from high-risk populations were aware of such risk. At present, however, there do not appear to be any published studies examining what variables actually predict healthy behaviors amongst the general public in Malaysia (i.e., those not diagnosed with a chronic disease). Thus, this study looks at the degree to which Malaysians are aware of diabetes and its risk factors and to what degree this and other variables such as family history of diabetes relate to lower risk lifestyles.

Based on the above, several research questions were formulated.

(a) Do individuals with better knowledge of type 2 diabetes engage more in healthy lifestyle behaviors?
(b) Does having a family history of type 2 diabetes increases one’s health knowledge?
(c) Are there any differences in terms of healthy lifestyle behaviors among people with and without family history of diabetes?
(d) Are there any ethnicity differences in terms of health knowledge and healthy lifestyle behaviors?
(e) Are there any relationships between perceived locus of control, health knowledge and healthy lifestyle behaviors?

## MATERIALS AND METHODS

### DESIGN

This was a cross-sectional study. Self-report questionnaires were used to investigate various sociocultural groups’ understanding of diabetes risk factors, their feelings of control over personal health outcomes, and how these factors predict lifestyle behaviors and preventive practices.

### ETHICS

The design and procedure of this study was reviewed in advance and given full approval by the Monash University Human Research Ethics Committee (MUHREC: CF12/3382-2012001623).

### PARTICIPANTS

Prerequisites for participating in this study were being a resident of Malaysia, over 18 years of age, and having not been diagnosed with type 2 diabetes. 794 people completed the questionnaire, 24 of whom were later excluded for incomplete responses or having existing medical conditions. Thus, data from a total of 770 participants from three different states; Penang, Selangor, and Terengganu, is included in these analyses. Ages ranged from 18 to 77 years old (*M* = 30.69, SD = 13.03). There were 308 males and 462 females. All participants were Malaysian residents; 34.5% were ethnic Malays, 40.9% were ethnic Chinese, 19.4% were ethnic Indians, and 3.8% were of other, or undefined, ethnicity (see **Table 1**).

**Table 1:** Descriptive statistics of participants.

Variable	Frequency (%)	Mean age
***Gender***
Male	308 (40)	32.38
Female	462 (60)	29.56
***Nationality***
Malaysian	728 (94.5)	39.76
Non-Malaysian	40 (5.2)	29.88
***Race***
Malay	266 (34.5)	27.28
Chinese	315 (40.9)	29.65
Indian	149 (19.4)	39.69
Others	29 (3.8)	26.55
***Occupation***
Housewife	56 (7.4)	45.38
Student	185 (24.0)	20.59
Business Owner	58 (7.5)	42.00
Professional	44 (5.7)	34.32
Administrator	20 (2.6)	36.80
Sales/service	280 (36.4)	29.20
Others	116 (15.2)	35.74
***Education***
Primary school	45 (5.8)	40.73
High school	386 (50.1)	32.31
Pre-university	137 (17.8)	29.25
University degree and above	185 (24.0)	26.06
Others	11 (1.4)	33.00
***Family history of diabetes***
Yes	242 (31.4)	30.39
No	526 (69.3)	31.27

### MEASURES

#### Diabetes knowledge test (DKT; Fitzgerald et al., 1998)

In order to understand if family members of diabetics or members of different ethnosocial groups are more knowledgeable than others about diabetes mellitus, the DKT was used. The test consisted of 14-items testing general knowledge of diabetes. Questions relate to common diabetes-related knowledge such as “Which of the following is a ‘sugar free food’?” and “For a person in good control, what effect does exercise have on blood glucose?” A higher score on this test indicates a better level of understanding of the disease. The alpha coefficients for the general test and the insulin-use subscale indicate that both are reliable (α > 0.70).

#### Multidimensional health locus of control scale (MHLC; Wallston et al., 1978)

The MHLC was used to assess locus of control, or perceived level of personal control, over health-related behavior. It is an 18-item instrument that measures three dimensions of locus of control: internality of health locus of control; powerful-other locus of control; and chance locus of control. All 18 items are arranged on a 6-point Likert scale ranging from “strongly agree” to “strongly disagree.” Higher scores indicate stronger belief in the dimensions of locus of control. In previous studies the internal reliability (Cronbach’s α) for this scale ranges from 0.67 to 0.77 for all three dimensions. This scale also has good criterion validity, correlating with participants’ state of health.

#### Simple lifestyle indicator questionnaire (SLIQ; Godwin, Streight, Dyachuk, van den Hooven, Ploemacher, Seguin & Cuthbertson)

The SLIQ was used to assess the lifestyle of the participants. There are five domains that are assessed which are diet, activity, stress, smoking and alcohol consumption. This is a 12-item instrument. Three questions ask about how often participants consume leafy green vegetables, fruits, and high fiber cereals or grains. Three questions inquire about the frequency of various light, moderate, and vigorous physical activities. Three questions assess alcohol consumption. Two questions inquire about smoking history, and one about general stress levels. Activity, Diet and Stress are arranged as Likert scales. Alcohol consumption is based on the total number of drinks consumed per week and the smoking score is based on whether the participant is a current, past, or non-smoker. For the purpose of this study, raw scores from each of these categories were used for analysis. The test-retest reliability of the 12-items is between 0.63 and 0.97. The Cronbach alpha was 0.58 for the diet domain and 0.60 for the activity domain. There was a correlation coefficient of 0.77 between the scores of the participants and blinded raters.

#### Demographic information

Participant’s gender, age, ethnicity, educational level, family medical history, and general health management method was gathered. No personally identifiable information (e.g., name, IC number) will be collected to ensure the confidentiality of the participants and their responses. Participants will be given contact information for the researcher if they wish to be informed of the research results.

### PROCEDURE

Individuals who were over 18, living in Malaysia, with no previous diagnosis of type 2 diabetes were recruited at markets and shopping areas frequented by a broad swath of the general population. Because these areas serve as social centers of the community in Malaysia, this was deemed to be a convenient way of accessing participants from many different social and economic backgrounds. The researchers situated themselves at tables in well trafficked areas throughout the day for several days and spoke to potential participants about the research. Participants were paid RM20 for completing the survey.

Because data was collected from the same locations over a period of multiple days many participants returned with friends or relatives by as well. Thus, a kind of snowballing recruitment was also employed. Prior to answering the questionnaire, all participants read a statement of purpose and provided their consent to participate. To ensure that participants had not been previously diagnosed with type 2 diabetes, in addition to screening at the time of survey distribution an additional question was included in the demographics section asking if the participant had ever been diagnosed with a chronic disease – 11 participants were excluded from analysis because they answered yes to this question.

## RESULTS

Overall means and standard deviations for the measures evaluated are noted on **Table 2**.

**Table 2:** Descriptive statistics of measurements.

Variable	Mean	SD
Diabetes knowledge test (DKT)	6.20	2.148
Internal locus of control	20.49	5.813
Powerful-other locus of control	22.68	6.051
Chance locus of control	26.79	5.681
Diet	7.92	3.546
Activity	12.22	5.923
Stress	3.31	1.294
Smoking	0.386	0.6862
Alcohol consumption	0.57	1.938

### FAMILY HISTORY

*T*-tests showed significant differences between those with family history of diabetes and those without family history of diabetes in diabetes knowledge, *t*(766) = -5.932, *p*= 0.000 (refer **Table 3**). Those with a family history (*M* = 6.86, SD = 2.050) were significantly more knowledgeable about diabetes as compared to those without family history of diabetes (*M* = 5.90, SD = 2.124) with the moderate effect size estimate of Cohen’s *d* = 0.46. There was also a significant difference between those with family history of diabetes and those without family history of diabetes in diet scores, *t*(766) = -3.461, *p* = 0.000. Those with a family history (*M* = 8.61, SD = 3.368) controlled their diet more as compared to those without family history of diabetes (*M* = 7.61, SD = 3.576), small effect size estimate of Cohen’s *d* = 0.29. Besides, it was found that there was a significant difference between those with family history of diabetes and those without family history of diabetes in activity scores, *t*(766) = 3.658, *p* = 0.000. Those with a family history (*M*= 11.10, SD = 5.630) were less active as compared to those without family history of diabetes (*M*= 12.77, SD = 5.972), small effect size estimate of Cohen’s *d* = -0.29. Furthermore, it was found that there was a significant difference between those with family history of diabetes and those without family history of diabetes in life stress scores, *t*(756) = -3.095, *p*< 0.01. Those with a family history (*M*= 3.52, SD = 1.242) were more stressed as compared to those without family history of diabetes (*M*= 3.21, SD = 1.310) with the small effect size estimate of Cohen’s *d* = 0.24.

**Table 3:** Independent sample *t*-test comparing differences between those with and without family history of diabetes on diabetes knowledge, life style behaviors and stress.

Variable	Mean scores		
	With family history	Without family history	*t*(N)
DKT	6.86	5.90	-5.932 (766)**
Diet	8.61	7.61	-3.461 (766)**
Activity	11.10	12.77	3.658 (766)**
Life stress	3.52	3.21	-3.095 (757)**

**p < 0.01.

### ETHNICITY

One-Way Multivariate analysis of variance (MANOVA) showed significant differences between ethnicity in educational level *F*(3,729) = 4.29, *p*< 0.01, diabetes knowledge, *F*(3,729) = 18.32, *p*< 0.001, diet, *F*(3,729) = 8.51, *p*< 0.001, activity level, *F*(3,729) = 3.16, *p*< 0.05, alcohol consumption, *F*(3,729) = 16.67, *p*< 0.001, and stress level, *F*(3,729) = 3.20, *p*< 0.05 (**Table 4**).

**Table 4:** One-way multivariate analysis of variance of education level, diabetes knowledge, diet, activity level, alcohol consumption, and life stress by ethnicity.

Variables	*SS*	*df*	*MS*	*F*	*p*
Educational level	10.86	3	3.62	4.29	0.005
Diabetes knowledge	238.37	3	79.46	18.32	0.000
Diet	314.01	3	104.67	8.51	0.000
Activity level	331.68	3	110.56	3.16	0.024
Alcohol consumption	173.92	3	57.96	16.67	0.000
Life Stress	15.90	3	5.30	3.20	0.023

Tukey *post hoc* comparison showed that Malay’s (*M* = 2.75, SD = 0.93) educational level was significantly higher than those of other races (*M* = 2.17, SD = 0.89). For diabetes knowledge, Indians (*M* = 7.04, SD = 2.24) had significantly better knowledge than Chinese (*M* = 5.62, SD = 2.12) and other races (*M* = 5.78, SD = 1.70) whereas Malay (*M* = 6.54, SD = 1.97) had significantly higher knowledge than Chinese (*M* 5.62 = , SD = 2.12), with medium partial squared effect size = 0.071. In terms of diet, Indian (*M* = 9.27, SD = 3.64) had significantly better diet control compared to Chinese (*M* = 7.59, SD = 3.30) and Malay (*M* = 7.73, SD = 3.68) with small partial squared effect size = 0.032. In terms of alcohol consumption, other races (*M* = 2.65, SD = 3.97) significantly consumed more alcohol than Malay (*M* = 0.12, SD = 0.80), Chinese (*M* = 0.82, SD = 2.17), and Indian (*M* = 0.44, SD = 2.01). Chinese (*M* = 0.82, SD = 2.17) also consumed significantly higher than Malay (*M* = 0.12, SD = 0.80) with medium partial squared effect size = 0.094. In terms of life stress, Chinese (*M* = 3.45, SD = 1.33) had significantly higher stress compared to Malay (*M* = 2.16, SD = 1.23) with small partial squared effect size = 0.018. (See **Table 5**).

**Table 5:** Average scores for diabetes knowledge and lifestyle behaviors by ethnicity.

Race	Mean (SD)					
	Education level	Diabetes knowledge	Diet	Activity	Alcohol	Life stress
Malay	2.75(0.93)	6.54(1.97)	7.73(3.68)	12.74(5.85)	0.12(0.80)	3.16(1.23)
Chinese	2.59(0.91)	5.62(2.12)	7.59(3.30)	11.56(5.87)	0.82(2.17)	3.45(1.33)
Indian	2.52(0.94)	7.04(2.24)	9.27(3.64)	12.16(6.25)	0.44(2.01)	3.49(1.26)
Others	2.17(0.89)	5.78(1.70)	7.48(3.34)	14.57(5.09)	2.65(3.97)	3.22(1.54)

### KNOWLEDGE OF DIABETES AND HEALTHY LIFESTYLES BEHAVIORS

Pearson’s Correlation Coefficient was calculated for all relationships amongst the DKT, SLIQ subscales, MHLC subscales and education level (see **Table 6**). A positive correlation was found between the DKT and diet scores, *r* = 0.193 (*N* = 770), *p*< 0.01, indicating that the more knowledgeable people are about diabetes, the more they watch and control their diet. Results also showed a small, but significant, negative correlation between the DKT and activity scores, *r* = -0.107 (*N* = 770), *p*< 0.01, indicating that more knowledgeable people engaged in less physical activity. There was also a negative correlation between the DKT and alcohol consumption, *r* = -0.107 (*N* = 769), *p*< 0.01, and a positive correlation between the DKT and life stress, *r* = 0.089 (*N* = 761), *p*< 0.05. People who were more knowledgeable about diabetes drank less alcohol, but appeared to have more life stress. A significant positive relationship was also found between diet and activity scores, *r* = 0.155 (*N* = 770), *p*< 0.01, indicating that people who controlled their diet engaged in more physical activity. Results also showed a significant negative correlation between activity level and life stress, *r*= -0.132 (*N* = 761), *p*< 0.01. The more people engaged in physical activity, the less stress they experienced.

**Table 6:** Correlation between DKT, SLIQ, MHLC subscales, age, education, and locus of control.

Variable	Diet	Activity	Alcohol	Stress	Age	Education	Internal	Chance	Powerful-others
DKT	0.193**	-0.107**	-0.107**	0.089*	0.107**	0.250**	0.245**	-0.447**	-0.111**
Diet		0.155**	-0.039	0.029	0.051	0.152**	0.073*	-0.147**	0.050
Activity			0.067	-0.132**	-0.130**	-0.135**	-0.114**	0.074*	0.046
Alcohol				-0.056	0.029	-0.098**	-0.082*	0.057	0.030
Stress					-0.119**	0.216**	0.128**	-0.53	-0.083*
Age						-0.265**	0.085*	0.051	0.177**
Education							0.293**	-0.113**	-0.196**
Internal								-0.537**	-0.515**
Chance									-0.447**

*p< 0.05, **p< 0.01.

### EDUCATION LEVEL

Results showed positive correlations between education and diabetes knowledge, *r*= 0.250 (*N* = 770), *p*< 0.01, diet, *r*= -0.152 (*N* = 770), *p*< 0.01, and life stress, *r*= 0.216 (*N* = 761), *p*< 0.01. Thus Malaysians with higher levels of education, tended toward greater diabetes knowledge, eating healthier and experiencing higher levels of stress. On the contrary, significant negative correlations were found between education level, and activity level, *r*= -0.135 (*N* = 769), *p*< 0.01, and alcohol consumption, *r*= -0.098 (*N* = 761), *p*< 0.01. In other words, more educated people engaged in less physical activity and drank alcohol somewhat less.

### AGE

Positive correlations were found between age and diabetes knowledge, *r*= 0.107 (*N* = 769), *p*< 0.01, internal locus of control, *r*= 0.085 (*N* = 769), *p*< 0.05, and powerful-others locus of control, *r*= 0.177 (*N* = 769), *p*< 0.01. Thus, older Malaysians’ diabetes knowledge, internal locus of control, and powerful-others locus of control tend be higher. Significant negative correlations were also found between age and activity, *r*= -0.130 (*N* = 769), *p*< 0.01, as well as stress level, *r*= -0.119 (*N* = 769), *p*< 0.01, indicating that older Malaysians tend engage in less physical activity, and experience less stress.

### PERCEIVED LOCUS OF CONTROL

There were significant positive correlations between internal locus of control and diabetes knowledge, *r*= 0.245 (*N* = 729), *p*< 0.01, diet control, *r*= 0.073 (*N* = 729), *p*< 0.05, and stress level, *r*= 0.128 (*N* = 729), *p*< 0.01. This suggests that Malaysians with higher internal locus of control tend toward greater diabetes knowledge, healthier diets, and have higher stress level. Results also showed that there are significant negative correlations between internal locus of control and physical activity level, *r*= -0.114 (*N* = 729), *p*< 0.01, and alcohol consumption, *r*= -0.082 (*N* = 729), *p*< 0.05. This shows that people who have higher internal locus of control engaged less in physical activity and drink less alcohol.

Results also showed that there are significant positive correlations between chance locus of control and physical activity level, *r*= 0.074 (*N* = 729), *p*< 0.05. This illustrates that people who have higher chance locus of control engaged more in physical activity. Also, results showed significant negative correlations between chance locus of control and diabetes knowledge, *r*= -0.447 (*N* = 729), *p*< 0.01, and diet control, *r*= -0.147 (*N* = 729), *p*< 0.01. These findings indicate that people with higher chance locus of control had poorer diabetes knowledge and engaged less in diet control. No significant correlations reported between chance locus of control and alcohol consumption, *r*= 0.057 (*N* = 729), *p*> 0.05, and stress level, *r*= -0.53 (*N* = 729), *p*> 0.05.

Significant negative correlations were also found between powerful-others locus of control and diabetes knowledge, *r*= -0.111 (*N* = 729), *p*< 0.01, and stress level, *r*= -0.083 (*N* = 729), *p*< 0.01. No significant relationships were found between powerful-others locus of control and diet control, *r*= 0.050 (*N* = 729), *p*> 0.05, physical activity level, *r*= 0.046 (*N* = 729), *p*> 0.05, and alcohol consumption, *r*= 0.030 (*N* = 729), *p*> 0.05.

### PHYSICAL ACTIVITY AS PREDICTED BY AGE, FAMILY HISTORY AND EDUCATION

To further analyze which of the factors predict physical activity level, multiple linear regression analyses were conducted. The dependent variable was the physical activity level and the independent variables were age, family history, education level, diabetes knowledge, three dimensions of locus of control (i.e., internal, chance, and powerful). The results showed that all independent variables together significantly accounted 5.6% of the variance in predicting physical activity level, *F*(6,745) = 6.266, *p*< 0.01 (see **Table 7**). However, when controlling for the interactions among variable only age, existing family history and education remained significant predictors of physical activity. Thus, for this sample of Malaysians, older age, having family members with diabetes, and higher education levels related to lower levels of physical activity.

**Table 7:** Summary of multiple regression analysis for education, diabetes knowledge, family history, and locus of control in predicting activity level.

Variable	*B*	*SE B*	β
Age	-0.071	0.017	-0.147**
Family history	-1.165	0.470	-0.092**
Education	-0.887	0.257	-0.138**
Diabetes knowledge	-0.067	0.106	-0.024
Internal locus of control	-0.316	0.266	-0.049
Chance locus of control	-0.062	0.263	-0.010
Powerful others locus of control	0.062	0.265	0.011

**R^*2*^* = 0.040 (*p* < 0.01)*.

*p < 0.05, **p < 0.01.

## DISCUSSION

The overall aim of this study was to better understand the growing incidence of type 2 diabetes in Malaysia, and thus better focus prevention efforts, by looking at the correlates of healthy behaviors (i.e., diet, activity level, stress, and alcohol consumption) as well as health knowledge and locus of control. Specifically with the Malaysian context in mind, we were also interested in how various demographic factors such as education and ethnicity relate to risk, again with the hopes of better tailoring prevention efforts toward the needs of specific groups.

Findings showed that, regardless of family history, more knowledge about diabetes related to more consumption of healthy foods such as vegetables, fruits, and high fiber grains, as well as lower alcohol consumption. This jibes well with previous studies relating knowledge to better dietary practices (Salmiah and Kamaruzaman, 2009) and lack of knowledge to unhealthy eating (Schillinger et al., 2006). Surprisingly, however, these results also indicate that, in Malaysia, people who know more about diabetes engage in less physical activity and lead more stressful lives. Similarly, education level was positively correlated to diabetes knowledge and stress level, yet negatively correlated to physical activity level.

Findings on the perceived locus of control indicate that people who perceived control over their own behavior had more diabetes knowledge, better controlled their diet, and consumed less alcohol. However, they also had higher levels of stress and engaged in less physical activity. The same trend was observed in people who are more educated as discussed earlier. So, again, Malaysians who are more educated appear to have better knowledge about diabetes, eat healthier diets, and consume less alcohol. However, they are more stressed and less active physically. This partially contradicts previous findings of internal locus of control positively relating to healthy eating and exercise habits (Morowatisharifabad et al., 2009; Schurer et al., 2012). External health locus of control, as might be expected, related to lower health knowledge. Findings indicate that people who perceived that their health is a matter of chance or due to powerful others, tend to have poorer knowledge, and were less likely to eat well, although they did experience less stress.

In addition, our findings revealed significant differences between people with and without family history of diabetes in terms of their diabetes knowledge and healthy lifestyle behaviors. Specifically, people with family history of diabetes had better knowledge about diabetes, and had more healthy diets. This group of people generally consumed more vegetables, fruits, and high fiber grains. These results are consistent with the findings of previous studies on African American adults (Baptiste-Roberts et al., 2007) and Pakistanis (Rosal et al., 2011) indicating that people with a family history of diabetes were more likely to eat fruits and vegetables. However, again, inconsistent with previous studies (Baptiste-Roberts et al., 2007; Rosal et al., 2011), people with a family history of diabetes engaged less in physical activity compared to people without family history. Findings also revealed that family history related to greater stress, possibly due to caretaking responsibilities, but again this would require further, more detailed, exploration.

Significant ethnic differences in health knowledge, behavior, and stress levels were also evident. Indians and Malays had better knowledge compared to Chinese and other races. On the other hand, Chinese and Malays had better diets compared to Indians. Less healthy diets among Indians likely contributes to their previously noted higher risk for metabolic syndrome and obesity compared to all other groups (Tan et al., 2011; Wee et al., 2011; Rampal et al., 2012). Malays (who in Malaysia are Muslim and for whom alcohol is forbidden), predictably, consumed the least amount of alcohol among all ethnicities. Chinese and Indians had higher levels of stress compared to Malays. It should be noted here that the Indian sample was significantly older on average than the other groups, although it is not clear how this might have influenced the results. Possibly a more age-matched Indian sample would have been more physically active, but also age generally related to better diets so an age matched Indian sample may have exhibited even less healthy eating behaviors.

Thus, compared to other ethnicities, Malays generally had better diabetes knowledge and diet control, engaged in more physical activity, consumed less alcohol and experienced less stress. Chinese, on the contrary, had less knowledge on diabetes, engaged in less physical activity, experienced more stress, but had better diets. Indians, although they had better diabetes knowledge, ate less fruits, vegetables and healthy grains, drank more alcohol, and had higher stress levels. These findings suggest that cultural factors related to ethnicity, such as traditional diets and norms related to exercise and alcohol consumption, may play an important role in health-related behaviors. Such factors are likely as, or even more, important than knowledge *per se*. Future work in diabetes prevention should take care to be sensitive to such cultural factors and take them into consideration when designing and tailoring interventions for specific groups.

Using regression to control for the relations amongst age, family history, education level, diabetes knowledge, and perceived locus of control as predictors of physical activity level; only age, family history, and education remained significant predictors. Notably, and counter-intuitively in the case of family history and education, these were all negative relationships which together combined to predict 5.6% of the variance in activity. This certainly warrants further investigation. However, given the large amount of variance unaccounted for it will also be necessary to identify other factors which must be contributing to risk levels. For example, are suitable locations available for exercise? Are there perceptions that exercise is dangerous or time consuming or cultural norms which contradict outdoor activity? Is there status associated with being overweight or inactive? There are certainly multiple outside factors involved in this scenario that need to be accounted for. More sensitive, possibly qualitative, measures will need to be used to achieve a nuanced understanding of the many environmental factors surely at play in this scenario.

### LIMITATIONS AND FUTURE STUDIES

Several limitations in this study should be taken into account when interpreting these findings: the measurements used in this study did not measure the total amount of food and sugar consumed by the participants. Thus some dietary risk factors may not be accounted for. Similarly, the measure of physical activity used here did not distinguish between work-related physical activity and leisure-time activities, an important distinction if we want to understand how education and locus of control are relating to activity. Also, of course, the self-report measures used here could reflect various types of response bias. A longitudinal approach in which participants log their eating and exercise behaviors online in real-time (though the use of smartphone technology, for example) would provide a more accurate assessment of actual behaviors as well as provide data for additional analyses such as total caloric intake, types of foods consumed, and the timing and intensity of physical activity. Such data would also allow us to tease apart the relationships among knowledge, education, cultural factors, locus of control their effect on diet and exercise patterns. Such a real-time tracking system could also serve as the foundation for health intervention programs that provide customized advice and coaching for individuals looking to improve their health.

It is important to note that the data collected here is essentially from a convenience sample. So, although it does represent a broad cross-section of the population, it is not necessarily representative of the entire country. These participants being from more urban areas, on average, are probably somewhat wealthier, and more educated than those from many other areas. Also, being correlational, these data should not be interpreted to represent causal relationships. We do not, for example, have any information as to why individuals choose to exercise or not, or to eat healthily or not. Based on these data we can only speculate as to the actual nature of relationships between various socio-cultural factors and behavior. Additionally, given the relatively small effect sizes observed in these data, there are certainly many other forces within the Malaysian context that are contributing to the recent rapid rise in diabetes rates. Possibly more exploratory approaches such as qualitative interviews and open-ended questions could help to uncover other more central factors underlying this problem.

## CONCLUSION

This research provides some initial guidance as to how education, locus of control, knowledge about diabetes, family history of diabetes, and ethnicity relate to healthy behaviors in the Malaysian context. Generally, as would be expected, education, internal locus of control, and knowledge were related to healthier eating patterns. However, the results related to exercise were somewhat contradictory to expectations; with higher education levels and family history correlating with lower levels of physical activity. Less surprising were findings that those with diabetic family members and those with higher internal control reported greater life stress. Ethnicity also seemed to play a role, especially in relation to diet: for example, Indians tended to have worse diets than other groups. Chinese diets were better than some others, but they exercised the least of all groups, and along with Indians, reported higher stress levels. Future research and intervention programs should take these cultural factors into account. Perhaps most important for understanding the roots of the diabetes epidemic in Malaysia, however, will be deciphering the negative correlations of education and family history to exercise. Is this simply a reflection of occupational differences between the more and less educated individuals (e.g., Duncan et al., 2010), or does it relate to a cultural narrative that equates higher status with a more sedentary lifestyle? Anecdotal evidence suggests it may be a combination of both factors, in which case it will be important to gear future interventions toward creating a cultural environment that places a higher value on physical activity. In future studies, more specific, possibly real-time, data on eating and exercise habits as well as qualitative reports detailing people’s attitudes toward, and beliefs about, exercise will help to clarify these issues. Again, such future investigations should be sure to include more exploratory, qualitative elements aimed at uncovering contributing factors not accounted for here. These data provide some initial insights, however, a great majority of the variance in risk-related behavior still remains unaccounted for. Future studies thus should look closely at other variables specific to the Malaysian context such as underlying attitudes or environmental factors that may be relating to behavior. Identifying such additional variables will be a key to successful intervention strategies, and thus should be a main focus of future studies. Creating a broad picture of these contextual risk factors will help create the most effective intervention strategies aimed at both the individual and public policy levels.

## Conflict of Interest Statement

The authors declare that the research was conducted in the absence of any commercial or financial relationships that could be construed as a potential conflict of interest.
